# Development and validation of a preliminary multivariable diagnostic model for identifying unusual infections in hospitalized patients

**DOI:** 10.17305/bb.2024.10447

**Published:** 2024-10-01

**Authors:** Aysun Tekin, Mohammad Joghataee, Lucrezia Rovati, Hong Hieu Truong, Claudia Castillo-Zambrano, Kushagra Kushagra, Nasrin Nikravangolsefid, Mahmut Ozkan, Ashish Gupta, Vitaly Herasevich, Juan Domecq, John O’Horo, Ognjen Gajic

**Affiliations:** 1 Division of Nephrology and Hypertension, Department of Internal Medicine, Mayo Clinic, Rochester, MN, USA; 2 Department of Business Analytics and Information Systems, Auburn University, Auburn, AL, USA; 3 Division of Pulmonary and Critical Care Medicine, Department of Internal Medicine, Mayo Clinic, Rochester, MN, USA; 4 School of Medicine and Surgery, University of Milano-Bicocca, Milan, Italy; 5 Department of Anesthesiology and Critical Care Medicine, Mayo Clinic, Rochester, MN, USA; 6 Division of Infectious Diseases, Mayo Clinic, Rochester, MN, USA

**Keywords:** Atypical infections, diagnostic delay, diagnostic model, multivariable model, rare infections

## Abstract

Diagnostic delay leads to poor outcomes in infections, and it occurs more often when the causative agent is unusual. Delays are attributable to failing to consider such diagnoses in a timely fashion. Using routinely collected electronic health record (EHR) data, we built a preliminary multivariable diagnostic model for early identification of unusual fungal infections and tuberculosis in hospitalized patients. We conducted a two-gate case-control study. Cases encompassed adult patients admitted to 19 Mayo Clinic enterprise hospitals between January 2010 and March 2023 diagnosed with blastomycosis, cryptococcosis, histoplasmosis, mucormycosis, pneumocystosis, or tuberculosis. Control groups were drawn from all admitted patients (random controls) and those with community-acquired infections (ID-controls). Development and validation datasets were created using randomization for dividing cases and controls (7:3), with a secondary validation using ID-controls. A logistic regression model was constructed using baseline and laboratory variables, with the unusual infections of interest outcome. The derivation dataset comprised 1043 cases and 7000 random controls, while the 451 cases were compared to 3000 random controls and 1990 ID-controls for validation. Within the derivation dataset, the model achieved an area under the curve (AUC) of 0.88 (95% confidence interval [CI]: 0.87–0.89) with a good calibration accuracy (Hosmer–Lemeshow *P* ═ 0.623). Comparable performance was observed in the primary (AUC ═ 0.88; 95% CI: 0.86–0.9) and secondary validation datasets (AUC ═ 0.84; 95% CI: 0.82–0.86). In this multicenter study, an EHR-based preliminary diagnostic model accurately identified five unusual fungal infections and tuberculosis in hospitalized patients. With further validation, this model could help decrease the time to diagnosis.

## Introduction

Guideline-based therapy and order sets have been indispensable in streamlining the management of infectious diseases [1]. Nevertheless, these tools have primarily been designed to address prevalent conditions. Less common pathogens not targeted by empiric guideline-based treatment are more likely to progress and need prompt diagnosis to optimize clinical care. However, even in regions where these pathogens are endemic, they are rarely prioritized in the differential diagnosis [2–4].

Numerous studies have highlighted notable delays in diagnosing patients with unusual pathogens [2, 3, 5, 6]. In our prior assessment of pulmonary blastomycosis patients at a large multisite medical center, we observed a considerable diagnostic delay, although 88% of the patients were diagnosed following the first-performed fungal test [7]. Our findings indicate that the main cause of the delay was the lack of timely consideration of blastomycosis. Similarly, a survey exploring perceived determinants of diagnostic delays in infections underscored the lack of timely consideration and appropriate testing as major contributors [8]. Despite advances in laboratory medicine’s increasing diagnostic capacity [9], their effectiveness hinges on the presumptive diagnosis of these entities in the appropriate clinical context.

Unusual infections require meticulous attention to a broad range of clinical variables. By employing an impartial approach, diagnostic models have the potential to identify characteristics often only recognized retrospectively as clues to an unusual diagnosis. Electronic health records (EHRs) capture an immense amount of real-time patient data, laying the foundation for models that can formulate inferences based on analysis of large quantity of data. Our objective was to develop and validate a preliminary diagnostic model using EHR data from Mayo Clinic Enterprise Hospitals located across the United States to identify patients hospitalized with specific unusual fungal infections (i.e., blastomycosis, cryptococcosis, histoplasmosis, mucormycosis, and pneumocystosis) or tuberculosis.

## Materials and methods

We adhered to the transparent reporting for individual prognosis or diagnosis (TRIPOD) recommendations (Table S1) [10].

### Study setting and participants

We employed a two-gate case-control design [11], in which patients with and without the disease were selected based on their disease status and tested, resulting in the score calculation being performed on two separate source populations. Subjects included adult patients admitted to Mayo Clinic enterprise hospitals, spanning three academic medical centers located in Minnesota, Florida, and Arizona, along with 16 community hospitals across Minnesota, Wisconsin, and Iowa. We excluded hospitalizations lasting less than 24 h and patients who opted out of participation in research.

Cases are defined as a group of unusual infections caused by infectious agents that fulfill all three criteria: (1) have the potential to cause severe systemic infection, (2) are not detectable by routine tests that are used for their typical associated infection foci, and (3) do not respond to recommended first-line empirical antimicrobials in terms of the drug or duration.

This analysis primarily focused on detecting blastomycosis, cryptococcosis, histoplasmosis, mucormycosis, pneumocystis, and tuberculosis. We exclusively screened diagnoses between January 2010 and March 2023 to mitigate the influence of diagnostic practice changes. To identify the cases, queries of International Classification of Diseases (ICD) codes were executed through the Mayo Data Explorer tool [12]. Afterward, physician–researchers (CCZ, NN, MO, LR, AT, HT) reviewed patient charts to confirm diagnoses.

The exclusion criteria included: lack of physician-confirmed diagnosis, latent or inactive infections, repeated hospitalizations, no hospitalization, and admission after more than two weeks of effective treatment.

We constructed two control datasets by screening patients between June 2018 and November 2022. Patients diagnosed with infections caused by pathogens that met our definition of unusual infections (Table S2) were excluded to prevent the inadvertent inclusion of cases in the control dataset. Given the objective of identifying unusual infections across all hospitalizations, the primary control dataset consisted of adult patients admitted on an urgent or emergent basis (i.e., random controls). We excluded the following hospitalizations from the control datasets: acute trauma-related admission (determined by ICD codes [13]), infection-related diagnoses leading to in-hospital mortality without a confirmed causative agent, readmissions.

As a secondary control dataset, we intended to assemble a dataset with admission characteristics comparable to our cases. Therefore, we evaluated patients diagnosed with community-acquired sepsis or septic shock, pneumonia, central nervous system infections, endocarditis, or infectious pericarditis with confirmed pathogens (determined by ICD codes) (i.e., ID-controls).

Control patients were randomly selected out of a large patient dataset for data collection.

### Outcomes

The outcome predicted by the model was the presence of infections of interest, determined through ICD codes and confirmed via chart reviews by researchers blinded to the candidate predictors.

### Predictor variables

We selected candidate variables based on a priori knowledge from a literature review of disease characteristics and expert opinion (OG, JO). We restricted the data variables to those objectively accessible through the EHR and available upon standard assessment of patients admitted on an urgent or emergent basis. Baseline variables were determined according to the status of individuals at the time of admission, while dynamic variables were limited to the initial 72 h of hospitalization. All variables evaluated for inclusion in the model and their definitions are outlined in Table S3. We conducted data collection in a blinded manner with respect to case or control statuses via queries over Mayo Data Explorer [12] and Intensive Care Unit Datamart [14] tools.

### Sample size

The number of variables to be tested was determined based on the rule of at least ten outcome events per variable [15]. Consequently, 104 variables were set as the cap for the model development phase, which included 1043 cases.

### Ethical statement

This study protocol along with the variable groups to be collected was reviewed and approved as a minimal risk study by Mayo Clinic Institutional Review Board (22-009881, approval date: 11/8/2022) under Common Rule 45 CFR 46.116. The requirement for written informed consent was waived.

**Figure 1.**
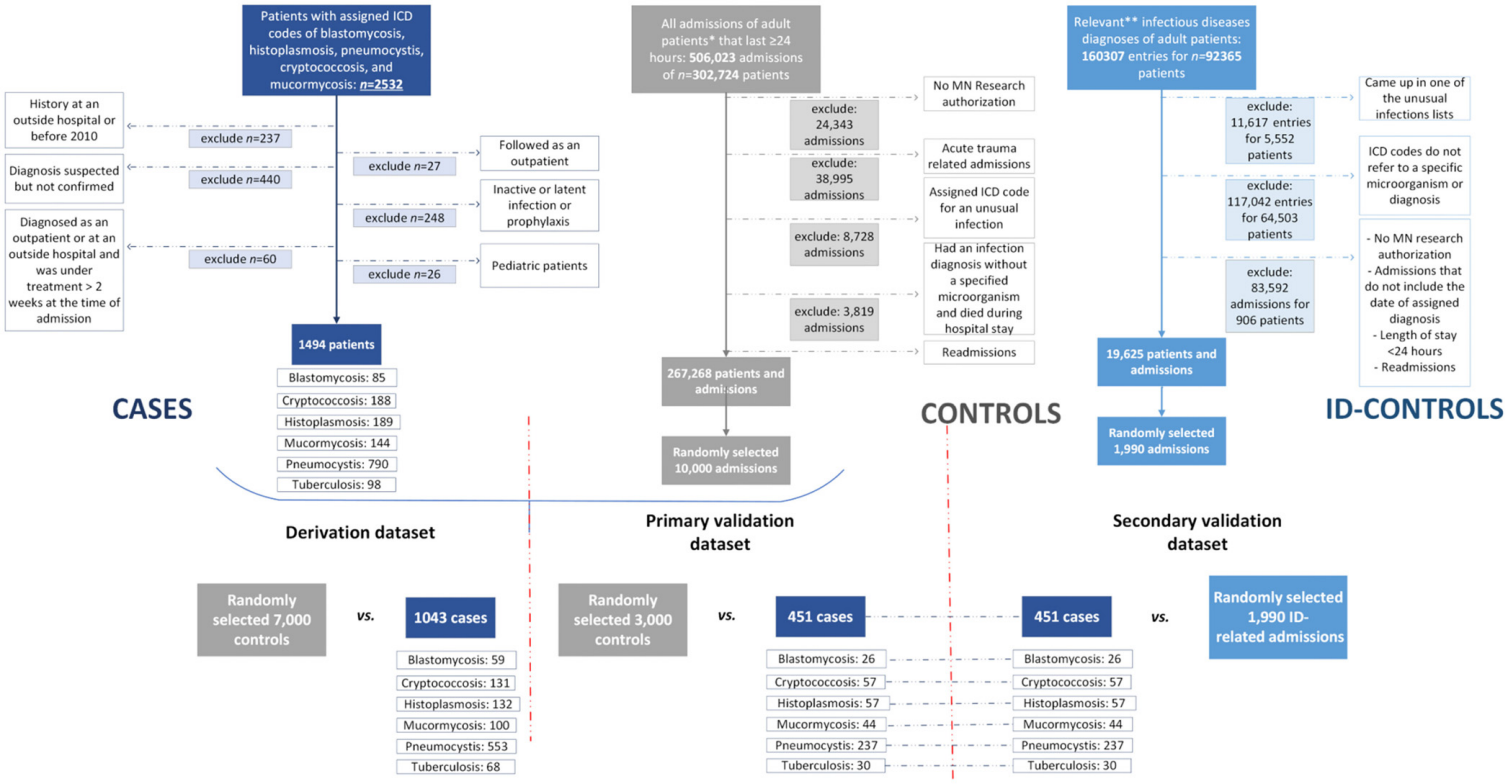
**Flowchart for the identification of the patients in derivation and validation datasets.** ICD: International Classification of Diseases; ID-controls: The control group that consisted of patients with community-acquired infectious diseases other than unusual infections.

### Statistical analysis

We described continuous data using the median and interquartile range (IQR), while presenting the categorical data as frequencies and percentages. Differences among case and control groups, as well as derivation and validation datasets, were evaluated via univariable analyses using chi-square and Mann–Whitney U tests. We randomly divided the dataset containing cases and random controls into derivation and validation subsets using a 7:3 ratio. To ensure a comparable distribution of individual unusual cases in both datasets, we stratified the 7:3 ratio application by the case type. We evaluated the missingness levels in the entire dataset before partitioning and excluded variables with over 35% missing values. We imputed the remaining variables via multivariate normal imputation, with a shrinkage estimator for covariances, creating a complete dataset.

To determine the linearity of patterns, we investigated the relationship between candidate predictors and the outcome using a Lowess smoothing approach. A multivariable binary logistic regression model was set, with the outcome representing either a case or control. Variables indicating the same domains were excluded from the model based on their relative importance. We employed a backward elimination variable selection method, guided by the Akaike information criterion. We examined the collinearity among included variables using variance inflation factors (VIFs). After evaluating all input, the final model was built. We assessed the calibration using the Hosmer–Lemeshow goodness of fit test [16]. Model performance was assessed using C-statistics by receiver operating characteristic curve plotting and area under the curve (AUC) calculation with corresponding 95% confidence intervals (CIs). We calculated the predicted probability using estimates from the derivation model to be used in the validation tests. The primary validation compared the validation cases with random controls, while the secondary validation compared the same cases with ID-controls. Sensitivity, specificity, and likelihood ratios were calculated for varying threshold points across all three datasets. To determine the predictive values, we calculated the disease prevalence among cases admitted after June 2018 and control admissions, excluding readmissions.

We performed several additional analyses tests on the primary validation dataset including sensitivity analyses (treating missing variables as normal by substituting missing values with the average normal, full-case analysis, excluding imputed values, and excluding cases admitted before June 2018) and subgroup analysis (separate evaluations for each unusual infection category).

The logistic regression model was built and tested using JMP Pro 14.1.0 (SAS Institute Inc., Cary, NC, USA, 1989–2021) and IBM SPSS v27.0 (Statistical Package for Social Sciences, USA) software. The comparison of receiver operating characteristic curves was conducted via DeLong’s test [17] using MedCalc Statistical Software. All tests were two-sided with a statistical significance of *P* ≤ 0.05.

## Results

We evaluated 2532 patients with assigned ICD codes for one of the unusual infections of interest and confirmed 1494 during structured chart reviews (Figure S1). Ten thousand random controls and 1990 ID-controls were randomly selected from hospitalizations meeting inclusion criteria (Figure 1). For derivation, 1043 cases were compared to 7000 random controls, while 451 cases were compared to 3000 random controls for primary validation. The same 451 cases from the validation dataset were compared to 1990 ID-controls for secondary validation.

### Model development

Table 1 presents the distribution of all variables assessed for the model in the derivation dataset, while Table 2 displays both validation datasets. The development and primary validation datasets were largely balanced. The ID-validation dataset exhibited distinct characteristics compared to the derivation dataset. Table S4 presents variable distribution across different datasets.

**Table 1:** Baseline characteristics for derivation dataset (before imputation)

**Variables**	**Total (*N* ═ 8043)**	**Cases (*n ═* 1043)**	**Controls (*n* ═ 7000)**	***P* value**
Age, years, median (IQR)	65 (49, 76)	61 (48, 71)	65 (50, 77)	**<0.001**
Sex, no. (%)				**<0.001**
Female	3899 (48.5)	378 (36.4)	3521 (50.3)	
Male	4136 (51.5)	660 (63.6)	3476 (49.7)	
Race, no. (%)				**<0.001**
African American	391 (4.9)	67 (6.4)	324 (4.6)	
Asian	154 (1.9)	42 (4)	112 (1.6)	
White	7136 (88.8)	868 (83.3)	6268 (89.6)	
Others	355 (4.4)	65 (6.2)	290 (4.1)	
Ethnicity, no. (%)				**<0.001**
Hispanic or Latino	372 (4.6)	40 (3.8)	332 (4.7)	
Not Hispanic or Latino	7456 (92.8)	945 (90.7)	6511 (93.1)	
Others, unknown, or not applicable	207 (2.6)	57 (5.5)	150 (2.1)	
Quarter of admission, no. (%)				**<0.001**
January–March	1723 (21.4)	276 (26.5)	1447 (20.7)	
April–June	1750 (21.8)	232 (22.2)	1518 (21.7)	
July–September	2297 (28.6)	261 (25)	2036 (29.1)	
October–December	2273 (28.3)	274 (26.3)	1999 (28.6)	
Admission location, no. (%)				**<0.001**
Arizona	1406 (17.5)	182 (17.4)	1224 (17.5)	
Florida	1226 (15.2)	151 (14.5)	1075 (15.4)	
MCHS	2482 (30.9)	132 (12.7)	2350 (33.6)	
Rochester	2929 (36.4)	578 (55.4)	2351 (33.6)	
Admission source, no. (%)				**<0.001**
Another hospital or care facility	1802 (22.4)	246 (23.6)	1556 (22.2)	
Outpatient or emergency department	930 (11.6)	350 (33.6)	580 (8.3)	
Others or unknown	5311 (66)	447 (42.9)	4864 (69.5)	
Pre-hospital location home	5898 (73.3)	857 (82.2)	5041 (72)	**<0.001**
Transferred patient	1182 (20.3)	74 (30.2)	1108 (19.9)	**<0.001**
Country of residence, no. (%)				**<0.001**
United States or Canada	7990 (99.4)	1023 (98.2)	6967 (99.6)	
Others	49 (0.6)	19 (1.8)	30 (0.4)	
^ *^African Region	1 (2)	1 (5.3)	0	
^ *^ Eastern Mediterranean Region	29 (59.2)	10 (52.6)	19 (63.3)	
^ *^ Region of the Americas, other than the US and Canada	16 (32.7)	6 (31.6)	10 (33.3)	
^ *^ South-East Asian Region	2 (4.1)	2 (10.5)	0	
^ *^ Western Pacific Region	1 (2)	0	1 (3.3)	
RUCA codes, no. (%)				**<0.001**
Metropolitan area	5212 (64.9)	591 (56.8)	4621 (66.1)	
Micropolitan area	1100 (13.7)	171 (16.4)	929 (13.3)	
Small town	898 (11.2)	111 (10.7)	787 (11.3)	
Rural areas	783 (9.7)	152 (14.6)	631 (9)	
Not coded	38 (0.5)	16 (1.5)	22 (0.3)	
Body mass index, kg/m^2^, median (IQR)	27.7 (23.7, 32.7)	26.3 (22.9, 31.2)	27.9 (23.8)	**<0.001**
Smoking, no. (%)				**<0.001**
Active smoker	3173 (39.5)	331 (31.7)	2842 (40.6)	
Never or ex-smoker	4870 (60.5)	712 (68.3)	4158 (59.4)	
Alcohol use disorder, no (%)	1016 (12.8)	87 (8.3)	929 (13.3)	**<0.001**
Comorbidities, no. (%)				
AIDS	116 (1.4)	64 (6.1)	52 (0.7)	**<0.001**
Asthma	2013 (25)	167 (16)	1846 (26.4)	**<0.001**
Cancer	2939 (36.5)	522 (50.1)	2417 (34.5)	**<0.001**
Cardiovascular disorders	2022 (25.1)	180 (17.3)	1842 (26.3)	**<0.001**
Chronic heart failure	2119 (26.3)	235 (22.5)	1884 (26.9)	**0.003**
Chronic kidney diseases	2440 (30.3)	305 (29.2)	2135 (30.5)	0.410
Chronic obstructive pulmonary disease	1668 (20.7)	212 (20.3)	1456 (20.8)	0.736
Connective tissue disease	514 (6.4)	63 (6)	451 (6.4)	0.625
Dementia	872 (10.8)	88 (8.4)	784 (11.2)	**0.007**
Diabetes	3229 (40.2)	416 (39.9)	2813 (40.2)	0.872
Dialysis	448 (5.6)	72 (6.9)	376 (5.4)	**0.044**
Hypertension	5314 (66.1)	591 (56.7)	4723 (67.5)	**<0.001**
Immunodeficiency	773 (9.6)	236 (22.6)	537 (7.7)	**<0.001**
Interstitial lung disease	2296 (28.6)	396 (38)	1900 (27.1)	**<0.001**
Leukemia	316 (3.9)	145 (13.9)	171 (2.4)	**<0.001**
Liver failure	2202 (27.4)	247 (23.7)	1955 (27.9)	**0.004**
Lymphoma	405 (5)	190 (18.2)	215 (3.1)	**<0.001**
Myocardial infarction	1447 (18)	120 (11.5)	1327 (19)	**<0.001**
Peptic ulcer disease	771 (9.6)	93 (8.9)	678 (9.7)	0.431
Peripheral vascular disease	2480 (30.8)	250 (24)	2230 (31.9)	**<0.001**
Valvular dysfunction	2595 (32.3)	315 (30.2)	2280 (32.6)	0.127
Laboratory variables at the time of admission, median (IQR)				
Hemoglobin, gr/dL	12.2 (10.2, 13.7)	10.4 (8.8, 12.2)	12.4 (10.5, 13.9)	**<0.001**
Hematocrit, %	37.5 (32.2, 41.7)	32.1 (27.5, 37.2)	38.1 (33.2, 42.1)	**<0.001**
Platelets, ×10(9)/L				
Highest	226 (169, 289)	186 (108, 279)	229 (175, 290)	**<0.001**
Lowest	222 (166, 285)	181 (102, 273)	226 (173, 286)	**<0.001**
Leukocytes, ×10(9)/L				
Highest	8.9 (6.5, 12.2)	7.6 (4.5, 11.9)	9 (6.7, 12.3)	**<0.001**
Lowest	8.7 (6.3, 11.8)	7.4 (4.3, 11.6)	8.8 (6.5, 11.9)	**<0.001**
Lymphocytes, ×10(9)/L				
Highest	1.18 (0.71, 1.79)	0.7 (0.4, 1.33)	1.24 (0.77, 1.83)	**<0.001**
Lowest	1.16 (0.69, 1.76)	0.69 (0.38, 1.32)	1.21 (0.75, 1.8)	**<0.001**
Neutrophils, ×10(9)/L				
Highest	6.29 (4.2, 9.6)	5.49 (2.91, 9.28)	6.38 (4.37, 9.65)	**<0.001**
Lowest	6.15 (4.11, 9.3)	5.16 (2.56, 8.87)	6.26 (4.28, 9.38)	**<0.001**
Monocytes, ×10(9)/L				
Highest	0.67 (0.46, 0.93)	0.54 (0.27, 0.84)	0.68 (0.48, 0.94)	**<0.001**
Lowest	0.65 (0.45, 0.91)	0.51 (0.26, 0.82)	0.66 (0.47, 0.92)	**<0.001**
Eosinophil, ×10(9)/L				
Highest	0.07 (0.01, 0.17)	0.03 (0, 0.11)	0.08 (0.02, 0.17)	**<0.001**
Lowest	0.07 (0.01, 0.16)	0.03 (0, 0.11)	0.07 (0.01, 0.17)	**<0.001**
Glucose, mg/dL				
Highest	123 (104, 162)	122 (102, 164)	123 (105, 162)	0.372
Lowest	123 (104, 161)	120 (101, 156)	123 (105, 162)	**0.002**
Lactate, mmol/L	1.6 (1.12, 2.4)	1.68 (1.2, 2.5)	1.6 (1.1, 2.4)	**0.033**
Creatinine, mg/dL	0.96 (0.77, 1.31)	0.92 (0.73, 1.30)	0.96 (0.77, 1.31)	0.161
Blood urea nitrogen, mg/dL	18 (13, 27)	19 (13, 28.1)	18 (12.9, 27)	**0.046**
Potassium, mmol/L				
Highest	4.2 (3.8, 4.5)	4.2 (3.8, 4.5)	4.1 (3.8, 4.5)	0.790
Lowest	4.1 (3.8, 4.4)	4.1 (3.7, 4.4)	4.1 (3.8, 4.4)	0.550
Sodium, mmol/L				
Highest	138 (135, 140)	136 (133, 139)	138 (135, 140)	**<0.001**
Lowest	137 (134, 140)	136 (133, 139)	138 (135, 140)	**<0.001**
Calcium, mmol/L				
Highest	9.1 (8.7, 9.5)	8.8 (8.3, 9.3)	9.2 (8.7, 9.5)	**<0.001**
Lowest	9.1 (8.6, 9.5)	8.8 (8.2, 9.3)	9.1 (8.7, 9.5)	**<0.001**
Bicarbonate, mmol/L	24 (22, 26)	24 (21, 26)	24 (22, 26)	**0.011**
Chloride, mmol/L				
Highest	101 (98, 104)	100 (97, 103)	101 (98, 104)	**<0.001**
Lowest	101 (97, 103)	100 (96, 103)	101 (97, 104)	**<0.001**
AST, U/L	28 (21, 46)	33 (22, 51)	28 (20, 45)	**<0.001**
ALT, U/L	23 (15, 41)	29 (18, 51)	22 (15, 39)	**<0.001**
ALP, U/L	90 (69, 128)	93 (69, 144)	89 (69, 125)	**0.016**
Total bilirubin, mg/dL	0.5 (0.3, 0.9)	0.5 (0.4, 0.9)	0.5 (0.3, 0.9)	0.399
Albumin, g/dL	3.7 (3.3, 4.1)	3.2 (2.8, 3.7)	3.8 (3.3, 4.2)	**<0.001**

Bold indicates statistical significance. ^*^Among those who reside outside of the United States or Canada. AIDS: Acquired immunodeficiency syndrome; ALP: Alkaline phosphatase; ALT: Alanine transaminase; AST: Aspartate aminotransferase; g/dL: Grams per deciliter; IQR: Interquartile range; MCHS: Mayo Clinic Health System; mg/dL: Milligrams per deciliter; mmol/L: Millimoles per liter; RUCA: Rural–urban commuting area; U/L: Units per liter.

**Figure 2.**
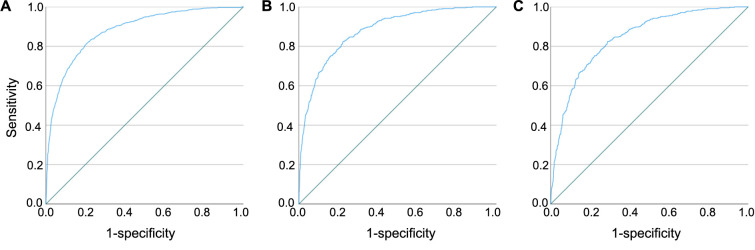
**Receiver operating characteristic curves for the model for detection of patients with unusual fungal infections and tuberculosis in derivation and validation cohorts.** (A) Model performance in the derivation dataset; the AUC was 0.88 (95% CI: 0.87–0.89); (B) Model performance in the primary validation dataset, compared to random controls; the AUC was 0.88 (95% CI: 0.86–0.9); (C) Model performance in the secondary validation dataset, compared to patients with infections; the AUC was 0.84 (95% CI: 0.82–0.86). AUC: Area under the receiver operating characteristic curve.

**Table 2:** Baseline characteristics for validation dataset (before imputation)

**Variables**	**Cases (*n ═* 451)**	**Random controls (*n* ═ 3000)**	*P* value^*^	**ID-controls (*n* ═ 1990)**	*P* value^**^
Age, years, median (IQR)	62 (49, 71)	65 (48, 76)	**<0.001**	76 (66, 85)	**<0.001**
Sex, no. (%)			**<0.001**		**<0.001**
Female	168 (37.4)	1527 (50.9)		942 (47.3)	
Male	281 (62.6)	1472 (49.1)		1048 (52.7)	
Race, no. (%)			**0.010**		**<0.001**
African American	31 (6.9)	122 (4.1)		21 (1.1)	
Asian	19 (4.2)	79 (2.6)		7 (0.4)	
White	379 (84)	2655 (88.5)		1920 (96.5)	
Others	22 (4.9)	144 (4.8)		42 (2.1)	
Ethnicity, no. (%)			0.557		**<0.001**
Hispanic or Latino	21 (4.7)	151 (5)		22 (1.1)	
Not Hispanic or Latino	416 (92.2)	2778 (92.7)		1936 (97.3)	
Others, unknown, or not applicable	14 (3.1)	69 (2.3)		32 (1.6)	
Quarter of admission, no. (%)			**<0.001**		**<0.001**
January–March	127 (28.2)	604 (20.1)		439 (22.1)	
April–June	100 (22.2)	677 (22.6)		703 (35.3)	
July–September	111 (24.6)	852 (28.4)		471 (23.7)	
October–December	113 (25.1)	867 (28.9)		377 (18.9)	
Admission location, no. (%)			**<0.001**		**<0.001**
Arizona	76 (16.9)	526 (17.5)		115 (5.8)	
Florida	75 (16.6)	417 (13.9)		60 (3)	
MCHS	46 (10.2)	1086 (36.2)		619 (31.1)	
Rochester	254 (56.3)	971 (32.4)		1196 (60.1)	
Admission source, no. (%)			**<0.001**		**<0.001**
Another hospital or care facility	122 (27.1)	665 (22.2)		482 (24.2)	
Outpatient or emergency department	148 (32.8)	241 (8)		171 (8.6)	
Others or unknown	181 (40.1)	2094 (69.8)		1337 (67.2)	
Pre-hospital location home	364 (80.7)	2137 (71.2)		1253 (63)	
Transferred patient	31 (6.9)	528 (21.8)	**<0.001**	253 (12.7)	**<0.001**
Country of residence, no. (%)			0.142		**0.014**
United States or Canada	445 (98.9)	2984 (99.5)		1986 (99.8)	
Others	5 (1.1)	16 (0.5)		4 (0.2)	
^***^African Region	0	1 (6.3)		0	
^***^ Eastern Mediterranean Region	5 (100)	7 (43.8)		4 (100)	
^***^ Region of the Americas, other than the US and Canada	0	7 (43.8)		0	
^***^ South-East Asian Region	0	1 (6.3)		0	
RUCA codes, no. (%)			**0.001**		**<0.001**
Metropolitan area	264 (58.8)	1974 (65.9)		1250 (62.8)	
Micropolitan area	64 (14.3)	411 (13.7)		346 (17.4)	
Small town	58 (12.9)	340 (11.3)		208 (10.5)	
Rural areas	58 (12.9)	259 (8.6)		182 (9.1)	
Not coded	5 (1.1)	12 (0.4)		3 (0.2)	
Body mass index, kg/m^2^, median (IQR)	26 (22.8, 30.1)	28.1 (24.2, 33.1)	**<0.001**	28.1 (23.9, 33.3)	**<0.001**
Smoking, no. (%)			**<0.001**		0.097
Active smoker	146 (32.4)	1233 (41.1)		566 (28.4)	
Never or ex-smoker	305 (67.6)	1767 (58.9)		1424 (71.6)	
Alcohol use disorder	38 (8.4)	391 (13.0)	**<0.001**	290 (14.6)	**<0.001**
Comorbidities, no. (%)					
AIDS	31 (6.9)	24 (0.8)	**<0.001**	33 (1.7)	**<0.001**
Asthma	78 (17.3)	783 (26.1)	**<0.001**	758 (38.1)	**<0.001**
Cancer	217 (48.2)	969 (32.3)	**<0.001**	1012 (50.9)	0.294
Cardiovascular disorders	83 (18.4)	802 (26.7)	**<0.001**	856 (43)	**<0.001**
Chronic heart failure	96 (21.3)	793 (26.4)	**0.003**	914 (45.9	**<0.001**
Chronic kidney diseases	149 (33)	908 (30.3)	0.234	1009 (50.7)	**<0.001**
Chronic obstructive pulmonary disease	93 (20.7)	620 (20.7)	1.00	766 (38.5)	**<0.001**
Connective tissue disease	31 (6.9)	180 (6)	0.463	241 (12.1)	**<0.001**
Dementia	28 (6.2)	344 (11.5)	**<0.001**	545 (27.4)	**<0.001**
Diabetes	181 (40.2)	1196 (39.9)	0.886	1236 (62.1)	**<0.001**
Dialysis	36 (8)	152 (5.1)	**0.011**	94 (4.7)	**0.005**
Hypertension	266 (59.1)	1975 (65.8)	**0.005**	1708 (85.9)	**<0.001**
Immunodeficiency	105 (23.3)	224 (7.5)	**<0.001**	160 (8)	**<0.001**
Interstitial lung disease	160 (35.6)	825 (27.5)	**<0.001**	1048 (52.7)	**<0.001**
Leukemia	68 (15.1)	78 (2.6)	**<0.001**	64 (3.2)	**<0.001**
Liver failure	105 (23.3)	838 (27.9)	**0.041**	217 (10.9)	**<0.001**
Lymphoma	72 (16)	97 (3.2)	**<0.001**	93 (4.7)	**<0.001**
Myocardial infarction	66 (14.7)	560 (18.7)	**0.040**	565 (28.4)	**<0.001**
Peptic ulcer disease	49 (10.9)	288 (9.6)	0.399	398 (20)	**<0.001**
Peripheral vascular disease	112 (24.9)	929 (31)	**0.009**	1225 (61.6)	**<0.001**
Valvular dysfunction	140 (31)	946 (31.5)	0.834	991 (49.8)	**<0.001**
Laboratory variables at the time of admission, median (IQR)					
Hemoglobin, g/dL	10.4 (9, 12)	12.5 (10.7, 13.9)	**<0.001**	11.7 (10.1, 13.1)	**<0.001**
Hematocrit, %	32.2 (28.2, 36.9)	38.2 (33.6, 42.3)	**<0.001**	36.5 (32.2, 40.4)	**<0.001**
Platelets, ×10(9)/L					
Highest	180 (104, 260)	229 (178, 290)	**<0.001**	206 (155, 274)	**<0.001**
Lowest	176 (94, 254)	227 (174, 286)	**<0.001**	202 (150, 269)	**<0.001**
Leukocytes, ×10(9)/L					
Highest	7.7 (4.3, 11.5)	8.9 (6.7, 12.2)	**<0.001**	11.8 (8.1, 16.4)	**<0.001**
Lowest	7.4 (4.1, 11.1)	8.8 (6.6, 11.8)	**<0.001**	11.4 (7.8, 15.9)	**<0.001**
Lymphocytes, ×10(9)/L					
Highest	0.76 (0.42, 1.3)	1.25 (0.8, 1.83)	**<0.001**	0.94 (0.59, 1.39)	**<0.001**
Lowest	0.74 (0.4, 1.28)	1.22 (0.78, 1.8)	**<0.001**	0.91 (0.57, 1.36)	**<0.001**
Neutrophils, ×10(9)/L					
Highest	5.36 (2.83, 9.03)	6.32 (4.32, 9.42)	**<0.001**	9.47 (6.02, 13.94)	**<0.001**
Lowest	5.2 (2.69, 8.82)	6.2 (4.27, 9.22)	**<0.001**	8.82 (5.41, 13.15)	**<0.001**
Monocytes, ×10(9)/L					
Highest	0.48 (0.26, 0.78)	0.68 (0.49, 0.94)	**<0.001**	0.8 (0.51, 1.17)	**<0.001**
Lowest	0.46 (0.24, 0.74)	0.66 (0.48, 0.93)	**<0.001**	0.78 (0.48, 1.14)	**<0.001**
Eosinophil, ×10(9)/L					
Highest	0.03 (0, 0.12)	0.08 (0.02, 0.18)	**<0.001**	0.03 (0, 0.1)	**0.003**
Lowest	0.03 (0, 0.11)	0.08 (0.02, 0.17)	**<0.001**	0.02 (0, 0.09)	**0.006**
Glucose, mg/dL					
Highest	124 (103, 174)	123 (104, 161)	0.620	141 (115, 188)	**<0.001**
Lowest	119 (100, 163)	123 (104, 161)	0.060	141 (115, 188)	**<0.001**
Lactate, mmol/L	1.6 (1.18, 2.6)	1.6 (1.1, 2.4)	0.428	1.9 (1.3, 2.9)	**<0.001**
Creatinine, mg/dL	1 (0.78, 1.4)	0.95 (0.76, 1.26)	0.249	1.15 (0.86, 1.62)	**<0.001**
Blood urea nitrogen, mg/dL	20 (13, 31)	17.9 (12, 26)	**<0.001**	23 (16, 33)	**<0.001**
Potassium, mmol/L					
Highest	4.2 (3.8, 4.5)	4.1 (3.8, 4.5)	0.791	4.2 (3.8, 4.6)	0.150
Lowest	4.1 (3.7, 4.4)	4.1 (3.7, 4.4)	0.184	4.1 (3.7, 4.4)	0.545
Sodium, mmol/L					
Highest	136 (133, 139)	138 (135, 140)	**<0.001**	137 (134, 140)	**0.002**
Lowest	136 (132, 139)	138 (135, 140)	**<0.001**	136 (133, 139)	**0.032**
Calcium, mmol/L					
Highest	8.8 (8.3, 9.3)	9.2 (8.8, 9.5)	**<0.001**	9 (8.6, 9.4)	**0.002**
Lowest	8.7 (8.2, 9.2)	9.1 (8.7, 9.5)	**<0.001**	8.9 (8.4, 9.3)	**0.032**
Bicarbonate, mmol/L	23 (21, 26)	24 (22, 26)	**0.013**	23 (21, 26)	0.880
Chloride, mmol/L					
Highest	100 (97, 103)	101 (98, 104)	**<0.001**	99 (96, 103)	0.068
Lowest	99 (96, 102)	101 (97, 104)	**<0.001**	99 (95, 102)	0.052
AST, U/L	35 (22, 60)	27 (20, 43)	**<0.001**	29 (22, 48)	**0.021**
ALT, U/L	27 (17, 53)	23 (15, 38)	**<0.001**	23 (15, 38)	**<0.001**
ALP, U/L	99 (73, 155)	88 (68, 118)	**<0.001**	96 (74, 142)	0.525
Total bilirubin, mg/dL	0.5 (0.4, 0.9)	0.5 (0.3, 0.9)	0.262	0.7 (0.4, 1.1)	**<0.001**
Albumin, g/dL	3.2 (2.9, 3.6)	3.8 (3.4, 4.2)	**<0.001**	3.5 (3.1, 3.9)	**<0.001**

Bold indicates statistical significance. ^*^Cases vs controls, ^**^Cases vs ID-controls, ^***^Among those who reside outside of the United States or Canada, ^****^Outside of the United States or unknown. AIDS: Acquired immunodeficiency syndrome; ALP: Alkaline phosphatase; ALT: Alanine transaminase; AST: Aspartate aminotransferase; CHF: Chronic heart failure; g/dL: Grams per deciliter; ID-controls: The control group that consisted of patients with community-acquired infectious diseases other than unusual infections; IQR: Interquartile range; MCHS: Mayo Clinic Health System; mg/dL: Milligrams per deciliter; mmol/L: Millimoles per liter; RUCA: Rural–urban commuting area; U/L: Units per liter.

Stepwise variable evaluation for the model is shown in Table S3. The included variables’ multicollinearity was evaluated by VIF, all of which were less than 10. The final model, including 37 variables, has been reported in Table 3. The model calibration was good, with a Hosmer–Lemeshow *P* value of 0.623.

**Table 3:** Multivariate diagnostic model for unusual fungal infections and tuberculosis in the derivation dataset

	**Variable**	**Estimate**	**95% CI**	***P* value**
	Intercept	13.69	10.93, 16.45	**<0.001**
Quarter of admission, reference: October–December	January–March	0.24	0.1, 0.38	**<0.001**
	April–June	0.08	−0.07, 0.22	0.304
	July–September	−0.19	−0.32, −0.05	**<0.001**
Admission location, reference: Mayo Clinic Health System Hospitals	Rochester	0.35	0.22, 0.48	**<0.001**
	Florida	0.09	−0.09, 0.26	0.338
	Arizona	0.31	0.14, 0.48	**<0.001**
	Non-transferred patient	−0.32	−0.44, −0.19	**<0.001**
	Age	−0.02	−0.02, −0.01	**<0.001**
	Female sex	−0.29	−0.38, −0.21	**<0.001**
Race, reference: White	Others	−0.36	−0.65, −0.07	**0.014**
	Asian	0.65	0.3, 0.99	**<0.001**
	Black or African American	0.1	−0.18, 0.38	0.469
RUCA codes, reference: Rural areas	Not coded	0.27	−0.38, 0.92	0.414
	Metropolitan	−0.39	−0.59, −0.18	**<0.001**
	Micropolitan	0.003	−0.24, 0.24	0.981
	Small town	−0.26	−0.52, 0.004	0.054
	Never or ex-smoker	0.21	0.11, 0.3	**<0.001**
	No alcohol use disorder	0.13	−0.03, 0.29	0.100
Admission source, reference: Another hospital or care facility	Others or unknown	−0.48	−0.6, −0.36	**<0.001**
	Outpatient or emergency department	1.08	0.94, 1.22	**<0.001**
Comorbidities, reference: Having the specific disease	No myocardial infarction	0.16	0.03, 0.29	**0.016**
	No chronic heart failure	0.08	−0.03, 0.19	0.130
	No peripheral vascular diseases	0.26	0.15, 0.37	**<0.001**
	No chronic obstructive pulmonary disease	−0.33	−0.47, −0.2	**<0.001**
	No interstitial lung disease	−0.44	−0.54, −0.34	**<0.001**
	No asthma	0.31	0.18, 0.43	**<0.001**
	No connective tissue disease	0.13	−0.04, 0.3	0.136
	No diabetes	−0.16	−0.26, −0.06	**0.002**
	No liver failure	0.26	0.15, 0.37	**<0.001**
	No cancer	0.14	0.04, 0.24	**<0.001**
	No leukemia	−0.52	−0.69, −0.36	**<0.001**
	No lymphoma	−0.66	−0.81, −0.52	**<0.001**
	No AIDS	−0.91	−1.15, −0.67	**<0.001**
	No hypertension	0.12	0.02, 0.22	0.023
	No immunodeficiency	−0.45	−0.56, −0.33	**<0.001**
Laboratory variables at the time of admission	Glucose, lowest	0.002	−0.003, 0.0005	**<0.001**
	Creatinine	−0.15	−0.22, −0.09	**<0.001**
	Potassium, lowest	0.15	0, 0.31	0.056
	Sodium, highest	−0.03	−0.06, 0.0005	**0.046**
	Chloride, lowest	−0.02	−0.05, −0.0004	**0.046**
	ALP	−0.0009	−0.002, −0.0003	**0.007**
	Albumin	−1.01	−1.16, −0.85	**<0.001**
	Hematocrit	−0.04	−0.05, −0.03	**<0.001**
	Platelets, lowest	−0.001	−0.002, −0.0003	**<0.001**
	Leukocytes, lowest	0.01	−0.001, 0.02	0.093
	Monocytes, highest	−0.55	−0.79, −0.32	**<0.001**
	Eosinophil, highest	−0.33	−0.8, 0.13	0.161

Bold indicates statistical significance. AIDS: Acquired immunodeficiency syndrome; ALP: Alkaline phosphatase; CI: Confidence interval; RUCA: Rural–urban commuting area.

### Model performance

The model distinguished cases from controls in the derivation dataset with an AUC of 0.88 (95% CI: 0.87–0.89) (Figure 2A). It performed similarly in the primary and secondary validation datasets (AUC ═ 0.88; 95% CI: 0.86–0.9 and AUC ═ 0.84; 95% CI: 0.82–0.86, respectively) (Figure 2B and 2C). To determine the predictive values, we calculated the disease prevalence among cases admitted after June 2018 (*n ═* 601) and control admissions (*n ═* 288, 334). Accordingly, assuming a prevalence of 0.21%, the positive predictive value in the validation dataset for a cutoff of 0.13 would be 0.012 (95% CI: 0.011–0.013) with a negative predictive value of 0.999 (95% CI: 0.999–0.999). Model performance for different cutoff values is provided in Table S5.

### Subgroup and sensitivity analyses

**Figure 3.**
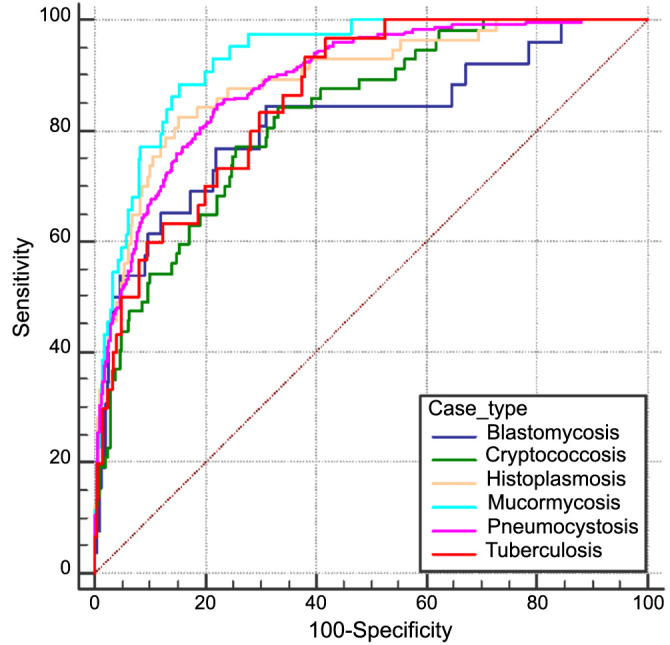
**Receiver operating characteristic curves for the model for detection of patients with specific unusual infections.** Model performance in detecting patients with blastomycosis vs random controls: AUC ═ 0.82 (95% CI: 0.72–0.92); Cryptococcosis vs random controls: AUC ═ 0.83 (95% CI: 0.78–0.88); Histoplasmosis vs random controls: AUC ═ 0.89 (95% CI: 0.85–0.94); Mucormycosis vs random controls: AUC ═ 0.93 (95% CI: 0.9–0.96); Pneumocystis vs random controls: AUC ═ 0.89 (95% CI: 0.87–0.91); Tuberculosis vs random controls: AUC ═ 0.86 (95% CI: 0.81–0.92). AUC: Area under the receiver operating characteristic curve.

In subgroup analyses evaluating model performance for individual diseases, the highest performance was observed among mucormycosis patients (AUC ═ 0.93; 95% CI: 0.9–0.96), whereas the lowest performance was observed for blastomycosis patients (AUC ═ 0.82; 95% CI: 0.72–0.92). Accordingly, the model performance for detecting mucormycosis was significantly higher than all other unusual infections except for histoplasmosis. Figure 3 depicts the results for all subgroups.

In sensitivity analyses considering all the missing variables as normal, the model’s discriminatory performance remained excellent with an AUC of 0.86 (95% CI: 0.85–0.88). Similarly, when the model was executed using a full-case approach, the AUC was 0.84 (95% CI: 0.78–0.89) (Figure S2A and S2B). Lastly, after excluding cases admitted before June 2018 (186 cases vs 3000 controls), the model discriminated the cases from controls with an AUC of 0.85 (95% CI: 0.83–0.88) (Figure S2C).

## Discussion

In this large multicenter retrospective study, we developed and validated a preliminary diagnostic model that distinguishes patients with five unusual fungal infections (i.e., blastomycosis, cryptococcosis, histoplasmosis, mucormycosis, and pneumocystosis) or tuberculosis from other hospitalizations with excellent performance. Our model relies on baseline variables and standard laboratory tests available in the EHR within the first three days of hospitalization without including any sophisticated microbiological or radiological evaluations. It consistently demonstrated strong performance in two separate validation sets, distinguishing cases from all hospitalizations and specifically from those admitted with other community-acquired infections. With further validation, both externally and prospectively, this model has the potential to become a supplementary tool to indicate patients who would benefit from additional microbiological evaluation or consultation with infectious disease specialists.

Advanced diagnostic tools are available for most pathogens included in this study [18–20], but their effectiveness relies on clinical suspicion. This poses a challenge due to the nonspecific presentation of these conditions [7, 8]. Accurate diagnosis requires timely recognition of complex patterns, which can be detected via a mathematical model. Many diagnostic and prognostic algorithms are more prominent in research settings than practical applications [21, 22]. This is partly because common conditions seldom necessitate advanced analytics. Conditions that tend to go unnoticed, however, such as unusual infections, are more appropriate targets because they require paying attention to many variables. Thus, diagnostic models may accelerate the diagnosis for unusual infections. Currently, no tools are available to aid medical teams in proactively considering these infections.

According to our model, the likelihood of infections of interest decreased with advancing age and among females. This is in line with the reported increased susceptibility of middle-aged males to some of these infections [23, 24]. Furthermore, Asian and Black or African American individuals exhibited an increased risk, consistent with surveillance studies [25, 26]. Rural living conditions are another established risk factor for unusual infections [27]. We evaluated this association using Rural–Urban Commuting Area codes classification in a simplified manner [28] and showed that inhabiting metropolitan areas displayed a lower probability of unusual infections than rural ones. Certain comorbidities like hypertension and chronic heart failure were linked to a reduced unusual infection risk, while conditions like diabetes, immunodeficiency, and pulmonary comorbidities, which are known risk factors, were associated with a higher probability [29–31]. For laboratory variables likely to be measured multiple times a day and those with potential clinical significance at both extremes, the highest and lowest recorded levels were evaluated. Notably, lower sodium levels were significantly associated with an increased risk of unusual infections, consistent with the well-established association between hyponatremia and granulomatous diseases [32–35].

This study employed a two-gate case-control approach, suitable for low-prevalence diseases but limited in terms of applicability of specificity to routine care [11]. To mitigate the study design’s impact, we utilized two distinct validation controls, i.e., random controls and individuals with community-acquired infections. Due to the extremely low prevalence of the infections of interest, the positive predictive values were low. Still, the model had acceptable accuracy across all three datasets, with high negative predictive values. The study results are promising in achieving high sensitivity, prompting plans for further validation through a prospective cohort study. The model’s complexity and reliance on estimates, rather than simplified calculations, pose challenges for bedside calculation. Instead, we envisioned this model as a readily calculated score within the EHR or alternative data visualization tools. To achieve this, we intend to leverage the existing control tower structure for Mayo Clinic enterprise hospitals [36]. This system will flag patients with high sensitivity. Given the low prevalence of the diseases and the control tower structure’s demonstrated efficiency in improving screening processes [37, 38], the expected workload will be manageable for one dedicated person to screen all flagged patients across the Mayo Clinic enterprise, even with low specificity. The specificity of the model will be gradually enhanced by incorporating feedback from the process.

The stepwise variable selection was essential to our model development. To handle missing data (when it was less than 35%), we opted for imputation, although it was not ideal. Unfortunately, this approach also prevented us from including potentially important information in our model if it was missing for more than 35% of the subjects. Still, as the availability of the included variables in the routine management of a patient admitted on an urgent or emergent basis was of utmost importance to this study in terms of determining the usability of the model, we opted for this approach. As laboratory tests are typically ordered based on clinical suspicion, a common score development approach is to treat missing data as normal [39]. To assess the viability of our model with such an approach, we repeated the validation process, treating missing values as normal, and the discriminatory capability remained excellent. We further tested the missing variables’ impact by running a sensitivity analysis solely on patients with complete data, yielding similar results. Therefore, the sensitivity analyses’ outcomes from our preliminary model are encouraging in terms of missing variables’ impact. Nonetheless, we recognize the need for further assessment of potentially significant variables which were overlooked due to the high missingness rates. These variables will be further evaluated during the prospective validation stage. The model’s performance to detect individual unusual infections was lowest for blastomycosis, as expected, given the lowest number of cases in the development dataset. Contrarily, the model performed best in detecting mucormycosis, although it was not the most prevalent in the development dataset. The accuracy of the model’s individual disease predictions warrants further exploration, as different models might be necessary to effectively predict individual infections.

One of this study’s strengths lies in its substantial sample size derived from a geographically diverse population of patients from academic and community hospitals. Another strength of our model is its consistent discriminatory performance across different datasets. Our investigation spanned a wide range of variables, including the highest and lowest values observed throughout the day, where both extremes could hold significance. The variables were selected considering their routine availability during hospital stays and ease of extraction from the EHR, excluding any complex tests or subjective evaluations to prioritize practicality. Additionally, all variables included are from the first 72 h of hospitalization, allowing the model to identify these patients early.

A primary limitation of this study is the utilization of an internal validation cohort, which potentially overestimates the model’s performance and restricts its applicability to broader populations. Therefore, the initial subsequent phase of this study will involve subjecting the preliminary model to external validation, aiming to provide a more accurate portrayal of its performance. Furthermore, the two-gate case-control design might have introduced spectrum bias, overestimating diagnostic performance [40]. This preliminary model needs to undergo testing in real-world settings, such as through prospective validation, before it might be considered suitable for clinical use. Additionally, we refrained from specifying a cutoff value for this model due to the constraints inherent in the study design, which needs to be addressed during the prospective validation phase. During the development of this preliminary model, certain significant factors, like pretest probability, were inadvertently overlooked. However, we intend to address this omission during the prospective validation phase, where we will explore their potential inclusion to fine-tune the model. Despite the large overall sample size, the number of cases in our dataset was small. To address this limitation, we intend to use techniques such as the Synthetic Minority Over-Sampling Technique algorithm to account for class imbalance in both the dataset at hand and subsequent validation processes. Moreover, some variables that could have had a significant impact on distinguishing infections of interest from other community-acquired infections were solely accessible in free text formats, which were not considered in this study. Additionally, the logistic regression model operates under the assumption of linearity among predictor variables, which may not always hold in practice. Incorporating additional machine learning techniques and potentially leveraging large language models in future stages of this study will help uncovering potential nonlinear relationships between predictors and outcomes, as well as incorporating other pertinent variables. Incorporating variables that are site-specific into the model and restricting the study to a single health system, albeit comprising a diverse range of hospitals, was another notable limitation diminishing the model’s generalizability, further stressing the imperative for external validation. Although the diseases fall under a common category in terms of typically requiring additional testing, their treatment approaches differ considerably. A multiclass prediction model that predicts specific classes of diseases will be the next step to pursue. Another limitation pertains to missing data. While the sensitivity analyses employing various approaches to manage missing data yielded promising results, further studies with more complete datasets are required. Excluding readmissions during the study period, as well as patients undergoing effective treatment for a certain period, may have introduced a sampling bias that could affect the outcomes of our assessment. However, these exclusions were considered essential to uphold the independence of observations and to target the early diagnosis of patients. Another limitation inherent to the retrospective design of the study was our dependence on ICD codes and chart reviews for confirming diagnoses. This prevented us from assessing the model’s impact on patients who were never accurately diagnosed. During the prospective validation phase, patients will be tracked in real time and confirmed by subject matter experts to mitigate the impact of this limitation. Furthermore, pediatric patients were outside of the scope of this study, restricting the relevance of the findings to the adult patient demographic. Lastly, because the preliminary diagnostic model was designed specifically for unusual infections, it would not capture the entire spectrum of infections in the population.

### Implications for practice and further research

Despite limitations, our study demonstrates the feasibility of a diagnostic framework to identify unusual infections, which are typically diagnosed late. The findings from this study will inform the development of EHR-based screening tools and bedside decision aids tasked at providing actionable information prompting appropriate evaluations. Thus, the diagnosis of unusual infections would be expedited, preventing adverse patient outcomes, unnecessary healthcare resource use, antibiotic resistance, and potential public health exposures. As the methodology primarily centers on detecting deviations from “typical”, i.e., indicating unusual conditions, it will also provide a framework that could be applicable to other rare diseases.

## Conclusion

In this large multicenter study, we developed and validated a model that accurately indicates unusual fungal infections and tuberculosis in hospitalized patients using readily available variables early during a hospitalization. The model also demonstrated excellent performance in distinguishing patients with unusual infections from those with other community-acquired infections. Based on routinely available EHR data, our model will inform the development of bedside tools for triggering evaluation for rare and unusual infectious diseases, thereby reducing the time to diagnosis.

## Supplemental data

Supplemental data are available online and can be accessed through the following link: https://www.bjbms.org/ojs/index.php/bjbms/article/view/10447/3242.

## Data Availability

The individual participant data that underlie the results reported in this article, after de-identification, and the study protocol are available to researchers who provide a methodologically sound proposal from the corresponding author at any time.
